# Health Care Professionals' Views on Discussing Sexual Wellbeing with Patients Who Have Had a Stroke: A Qualitative Study

**DOI:** 10.1371/journal.pone.0078802

**Published:** 2013-10-29

**Authors:** Ruth M. Mellor, Sheila M. Greenfield, George Dowswell, James P. Sheppard, Tom Quinn, Richard J. McManus

**Affiliations:** 1 Primary Care Clinical Sciences, University of Birmingham, Birmingham, United Kingdom; 2 Department of Primary Care Health Sciences, University of Oxford, Oxford, United Kingdom; 3 Faculty of Health and Medical Sciences, University of Surrey, Guildford, United Kingdom; Charité-Universitätsmedizin Berlin, Germany

## Abstract

**Objectives:**

To examine the experiences of health care professionals discussing sexual wellbeing with patients who have had a stroke.

**Design:**

In-depth qualitative interview study with purposive sampling and thematic analysis.

**Participants:**

30 health care professionals purposively recruited to include different roles and settings along the stroke patient pathway in secondary and primary care.

**Setting:**

Two hospitals and three general practices in the West Midlands, UK.

**Results:**

Sexual wellbeing was a topic that participants did not raise with patients and was infrequently raised by patients. Barriers to raising discussion were on four levels: structural, health care professional, patient, and professional-patient interface. Barriers within these levels included: sexual wellbeing not present within hospital stroke policy; the perception that sexual wellbeing was not within participants' role; participants' concern that raising the issue could cause harm to the patient; and the views that discussion would be inappropriate with older people or unimportant to women. Resources exist to aid discussion but many participants were unaware of them, and most of those that were, did not use them routinely.

**Conclusions:**

Participants lacked motivation, ownership, and the confidence and skills to raise sexual wellbeing routinely after stroke. Similar findings have been reported in cancer care and other taboo subjects such as incontinence potentially resulting in a sub-optimal experience for patients. Normalisation of the inclusion of sensitive topics in discussions post-stroke does not seem to need significant structural intervention and simple changes such as information provision and legitimisation through consideration of the issue in standard care policies may be all that is required. The experiences recounted by professionals in this study suggest that such changes are needed now.

## Introduction

Stroke is a common cause of morbidity and mortality with an incidence ranging from 95–269 per 100,000 population in Europe and the US[1], causing, in 2010, 11.1% of deaths globally[2]. One effect of stroke sometimes overlooked is the impact on the sex lives of stroke survivors and their spouses[3]. Whilst a minority of patients experience hypersexuality following stroke[4], hyposexuality is frequent with several international studies describing reduction in libido[5], [6]; reduced frequency[7], or cessation[5] of intercourse; reduced erectile capacity[5] or erectile dysfunction[8] for men; and reduced vaginal secretion for women[5]. Lowered sexual wellbeing is not exclusive to older people; middle aged stroke survivors[9] or spouses[10] can also be affected. Furthermore problems may persist, with only 41% of stroke survivors in the Netherlands reporting satisfaction with their sex life three years post-stroke[11]. Around 50% of patients and spouses reported interest in sexual counselling as part of rehabilitation[5], suggesting patients have a desire to know more.

There are several potential reasons for these issues: physical disability could prevent couples from practicing certain sexual positions. Communication problems may prevent discussions around sex, and even where speech is unaffected, it can be a difficult topic to raise[12], [13]. Co-morbidities or their associated medications may influence the problem, for example diabetes, heart disease[8], [14], or depression[6], [15].

Change in life context can influence sexual satisfaction and expectations. The move from ‘spouse’ to ‘caregiver’ influences relationship dynamics, roles and identities, which may now be less compatible with that of ‘lover’[13], [16]. Changes in physical appearance can affect self-consciousness and willingness to have physical contact[16] which may impact on patient and partner's view on their desirability[13]. Resulting lack of sex within the relationship can cause guilt and further stress[16]. A particular concern is that sex would lead to a further stroke[12], [13], [16].

The UK National Stroke Strategy states patients need access to emotional support services (which includes supporting sexual wellbeing) and that all staff working with stroke should be able to signpost relevant specialist help[17]. Resources regarding sex after stroke are available. For example, the Stroke Association has a specific ‘Sex after stroke’ information leaflet[18].

Health care professionals (HCPs) have reported difficulty discussing sexual wellbeing in other situations including cancer[19]–[21], cardiac patients[22], [23], the elderly[24], and lesbian or gay patients[25]. Barriers include the widely shared societal assumption that older people are not sexually active [26] HCPs also report a lack of time, belief that patients are too ill, assumptions that disfigured bodies are no longer attractive, concerns about uncovering needs which may not be addressed, fear of medicolegal boundaries and simply the presence of third parties during consultations [27] HCPs often report feeling inadequately trained or insufficiently skilled [19] However there is a dearth of information on HCPs' perspectives in relation to patients who have had a stroke, with only McLaughlin and Cregan's[28] survey of 13 stroke HCPs in Northern Ireland available. They suggested just over half thought addressing sexuality would be part of their rehabilitation role, although the majority had not received training in this area.

There are well known patient barriers to discussion of sexual wellbeing. Half of patients calling an erectile dysfunction helpline in Italy had not discussed it with their doctor[29]. Identifying which HCP to approach is problematic for many patients. A study of women with type two diabetes reported not feeling comfortable discussing sexual wellbeing with their GP, feeling a gynaecologist, if anyone, would be more appropriate[30]. In contrast, 78% of cardiac patients said of all sexual health services available to them, they would prefer to consult with a General Practitioner (GP)[31]. Almost half (48%) of cardiac patients would have liked chance to discuss, or extend discussions around sex[31]. Men with colorectal cancer[32] and women with ovarian cancer[33] reported difficulty raising the issue but wanted to discuss it. Barriers to older people raising sexual problems also include perceiving it as normal with aging, disassociating it from a health problem, not viewing the problem as serious, and concern that the physician would be uncomfortable discussing it[34]. Patients worried about recurrence of illness often perceive a high risk associated with potentially offending HCPs who are responsible for their care [32], [35]. Yet counselling around sexual activity can influence resumption of sex. Both male and female patients with acute myocardial infarction who had not received counselling on discharge reported greater loss of sexual activity 12 months later[36] than those who received counselling.

This research used a qualitative approach to examine the views and experiences of health care providers discussing sexual wellbeing with patients who have had a stroke to identify barriers and suggest improvements to information provision.

## Methods

The Consolidated criteria for reporting qualitative studies (COREQ) checklist [37] helped guide the reporting of this study.

### Ethics statement

This study was approved by the London-Queen Square Research Ethics Committee (09/H0716/71).

### Conducting interviews

In-depth interviews with HCPs who were involved in roles within the stroke patient pathway were conducted by the first author Ruth Mellor (RM). HCPs were asked about their experience of discussing sexual wellbeing with patients who have had a stroke and resources available to assist them. Within the study, sexual wellbeing was defined broadly, in line with the WHO[38] definition: ‘a state of physical, emotional, mental and social well-being related to sexuality; it is not merely the absence of disease, dysfunction or infirmity.’ However this definition was not imposed upon participants, who were free to raise and discuss whichever elements they wished.

The study was part of a programme evaluating current practice and studying service change within the Birmingham and Black Country Collaboration for Leadership in Applied Health Research and Care project[39]. Participants worked within study sites; two hospital trusts and three general practices in the West Midlands. A set of sexual wellbeing questions was embedded within a longer interview on their experience of the stroke patient pathway. Other topics within the longer interview included identification of stroke, participants understanding of the wider pathway and the linking up of services across the pathway.

HCPs were purposively recruited on location and role: across the stroke patient pathway from hospital admission, acute and rehabilitation wards, to care in the community; and targeting doctors, nurses, therapists, health care assistants (HCA), and support coordinators. A mixture of snowball (where participants informed their colleagues about the study and suggested they participate)[40] and judgement sampling (where participants were specifically selected from the total HCP population based on RM's judgement and experience of the study)[41] were used for recruitment. Prior to starting recruiting participants at the hospital trusts, gatekeepers (consultants on wards) gave permission for staff to be approached, but were not informed which staff would be approached or who agreed to be interviewed. RM had an honorary contract at both trusts and had been present on site prior to recruitment for other aspects of her job. She advertised the study at trust stroke training sessions and through face-to-face contact on the ward. RM is based within a Primary Care department and recruited participants from general practice through GP contacts within the department. Two participants agreed to be interviewed but then dropped out due to practical difficulties; however their location and role within the stroke pathway characteristics were covered by other participants.

Participants received an information sheet that stated the purpose of the interview: ‘to find out about the views of healthcare professionals on the service offered to patients by the Stroke and TIA services’. Prior to each interview RM introduced herself, her role in the CLARHC study, and answered any questions. If participants had queries about the study post-interview, the information sheet contained study contact details, including details for complaints.

Interviews continued to ask about the discussion of sexual wellbeing with patients post-stroke until saturation was reached, where no new themes emerged and collecting additional data on it seemed unnecessary. Within the wider study further interviews were conducted around other elements of the stroke patient pathway which did not ask about sexual wellbeing, some with staff such as paramedics and GP receptionists where the topics was not deemed relevant to their role, these findings will be reported elsewhere.

Interviews were conducted between September 2011 and December 2012, at participants' place of work. Interview duration ranged from 14 to 68 minutes, average 34 minutes, this included discussion of other topics, but excluded introductions and questions. All participants were interviewed once by RM, individually (21 participants), in pairs (six participants) or in one case in a three. Written informed consent was gained from all participants, which included consent for quotes, with name and identifier removed, to be used in publications. Interviews were audio recorded and transcribed verbatim. Fieldnotes were recorded after some interviews.

Data will not be deposited in a publically available resource, but the study will comply with University of Birmingham archiving policies.

### Analysis

Transcripts were checked for completeness and accuracy and analysed by RM (a non-clinical mixed methods sexual health researcher; she has experience and training in qualitative methods through completion of a mixed methods PhD). Identifiable information from transcripts i.e. names, hospital names, were removed. Data were managed using NVivo 9 (QSR International)[42]. An inductive, data rather than hypothesis driven, approach was taken. The sections of text concerning sexual wellbeing were extracted from the rest of the interview data and considered separately from the rest. After the first 10 interviews, initial analysis of the sexual wellbeing data was conducted using the ‘one sheet of paper’ (OSOP) method, where the issues raised in each extract, along with the participants pseudonym, were noted onto a single large sheet of paper[43]. These issues were grouped to show the main themes and to identify variation in responses. The OSOP highlighted further areas requiring discussion which were included in the topic guide, specifically ease of discussion in front of patients' relatives and HCP perception of safety of having sex post-stroke.

Additional themes/sub-themes were added when they emerged from later interviews[44]. A constant comparison approach, where different segments of data are compared to look for similarities and differences[45], was used in the analysis, at the individual level and also by role and stage in the stroke patient pathway.

To ensure rigour within the analyses and a multi-disciplinary perspective, the last author, Richard McManus, (a general practitioner) double coded a subset of interviews and checked analytical interpretations. Differences of interpretation were resolved by discussion. Transcripts were not returned to participants to get feedback on the findings; a lay summary report will be sent to those who requested one, at the end of the study. Excepting McLaughlin & Cregan's[28] 13 person survey, no qualitative evidence existed to test the qualitative components against.

## Results

Thirty HCPs were interviewed before saturation on the discussion of sexual wellbeing with patients theme was achieved: a range of HCPs were recruited, but most worked within secondary care, the biggest group was doctors, the majority were women, and approximately half had over five years of experience in their roles (Table 1).

**Table 1:** Characteristics of participants.

Category	Sub-category	N (N = 30)
Where in the pathway they work	Accident and Emergency Department	1
	In hospital – acute ward	12
	In hospital – rehabilitation	5
	Across hospital (i.e. accident and emergency department, acute ward, rehabilitation clinics)	5
	Primary care	3
	Across hospital and community	4
Position	Nurse	6
	Doctor	10
	Therapist	7
	Support coordinator	4
	Health care assistant	3
Gender	Male	9
	Female	21
Years of experience in the role	Less than 1 year	4
	Greater than or equal to 1 year, less than 5 years	11
	Greater than or equal to 5 years, less than 10 years	7
	10 years or more	7
	Did not answer	1

Their results are structured into three main themes two of which reflect the overarching topics raised in the sexual wellbeing section of the interview topic guide; experiences of discussing sexual wellbeing post stroke and the potential resources available to assist with these concerns. The third theme, barriers to discussion, emerged from the analysis. It was split into four sub-themes: the structural care pathway level, health care professional level, patient level, and HCP-patient interface. The emphasis on discussing barriers was chosen as through their identification and probing, solutions can be raised. Only the participant's occupational role is presented under each quotation to maintain anonymity.

### Experience of raising or having the issue of sexual well-being raised

Support coordinators were the only HCPs who reported raising the issue of sexual wellbeing with patients who have had a stroke but used their discretion as to when they thought this was appropriate. Other HCPs had either never thought about it, or did not think it was an appropriate subject for them to raise.

*Interviewer (I): Do you ever talk to your patients about sex? Sex and stroke, is it an issue?*

HCP7: I've never actually had a conversation about it [laugh], I've never thought about it to be honest. HCP7-Nurse

HCPs varied in the frequency with which they had experienced patients raise the issue themselves. Some had never had a stroke patient raise it, for others it was a rare occurrence (once or twice in career thus far) but for some HCPs it was an uncommon but not unusual discussion topic. It was more likely to be raised in a one-to-one setting, for example in a clinic appointment or at the patients' home, although one Health Care Assistant (HCA) reported a patient raising it whilst she escorted him to the toilet. Usually such discussions took place after the patient had left hospital as can be seen in HCP6's quotation.

…in their acute phase [in hospital], it doesn't come up that often. It's rarely a conversation that we have. But clearly l see patients after they've been discharged, approximately six weeks after they've gone home. Once you've gone home, settled back into home life, there then does come, this thought about lots and lots and lots of different things. Can I go and play cricket still, can I play football, blah blah blah, walking the dog, going on holiday, sex. All comes in, definitely. HCP6-Nurse

When sexual wellbeing was discussed, the reported topics were around: safety of having sex, timing of resuming sex, erectile problems, and at the other end of the spectrum, the normality of not wanting to have sex.

There was a sub-set of the HCPs, with little experience of discussing sex, who reported it in the context of people with mental health problems. In addition, one experienced participant (who described having many conversations around sexual wellbeing in a past role), voiced concerns about the appropriateness of patients raising discussion around sexual wellbeing with HCAs; that this could be a patient manipulating the HCA-patient relationship, rather than genuine information seeking behaviour. This association and the general undercurrents of not discussing sexual health highlights it remains a taboo topic.

HCP2: I've not had any patients ask for ages about that and if they have they've been - it's been in an inappropriate conversation, that the patient has asked…

HCP1: If they lose their inhibitions.

HCP2: Then they can sometimes talk about that kind of stuff. But actually they're probably not really under - sort of aware that they're asking those questions. So…

*I: Rather than…?*

HCP2: Yeah, rather than them actively going ‘oh what's, what's going to happen?’

HCP1&2-Therapists

HCP30: The fact that actually because you're dressing them, you're bathing them. Its hands on care, when people actually ask them, ‘Can I?’ or, ‘Do you think I should?’ I think there's a very - it's not a line to be drawn, but I think you actually have to be careful it's not all part and parcel of something else.

*I: That they're like trying to hit on the HCAs you mean?*

HCP30: Yeah, or, or to an extent - I was going to say groom, but that may be not the right word, but actually sort of start the conversation, start the ball rolling. HCP30-Doctor

### Barriers and opportunities to raising the issue

Barriers to the provision of information were divided into four sub-themes: the structural care pathway level, health care professional level, patient level, and HCP-patient interface. Each will be discussed in turn.

#### Structural care pathway level

At an organisational level, staff involved with hospital management perceived that sexual wellbeing was not an area of concern in the hospital stroke patient pathway, as it was not “included in the [hospital] stroke policy”. Moreover participants viewed there was lack of specialist service provision in this area, which was perceived in part to be due to lack of demand for such services.

I think you would need to draw on other services. It [discussing sexual wellbeing] wouldn't be a service that we would put in for stroke, but it would be a service that we would put in for a cohort of patients. So, for example like, on the neurology wards, clearly it's a much bigger problem, but can we draw from that service HCP17-Nurse

In contrast some of the support coordinators reported that sexual health was one of the areas specified in their assessment tool. This could legitimise such discussions, although time limitations on visits might curtail or prevent discussion.

In the first assessment tool that's [sexual health] one of the options… And our visits, well we try and keep them to under an hour; I think maximum of an hour and a half is plenty. But there's a lot to cover. So it might be that you feel ‘Well that's enough for now, we'll pick up on something a bit later if needs be’ HCP11-Support Coordinator

#### Health Care Professional level

At the individual HCP level, particularly within the hospital setting there was the perception that sexual wellbeing was not within their role and that someone else was better suited to deal with it.

I think doctors are probably not particularly…probably the nurse is more aware. The doctors are not very good at, not really interested in delving into that side of things HCP24-Doctor

Reasons given for non-inclusion in role included lack of training, a concern that if something was raised it would be of a magnitude that they were unable to deal with, although questions such as ‘will things go back to normal’ some HCPs were unsure how to answer. For some staff there was implicit embarrassment around dealing with the topic, for example six participants did not use the word ‘sex’ or a similar phrase during the interview, rather referring to ‘it’ and ‘those kinds of things’. This embarrassment was not systemic, as other staff in the hospital setting reported a willingness to have such discussions. However, concern was expressed about a lack of formal support nor any obvious referral pathway should problems be identified, nor had they seen colleagues engaged in such discussions. This reinforced the feeling that sexual issues were not within their remit – someone else's problem - and raising them could leave the hospital staff exposed.

I know OT [Occupational Therapist] wise we did do a bit about it at Uni [university]… like more so as something to, like, that could be looked at by an OT, but I don't know, I've never, anywhere that I've worked, we've necessarily, like, talked to patients about it, but I suppose we could, OT wise we could. It's an activity that you do, isn't it? HCP21-Therapist

For HCPs seeing patients when they had left hospital, the perspective was that patients were free to raise what they liked (including sexual wellbeing) with the acknowledgement that the HCP would deal with what they could and direct them to relevant services. The emphasis on passing patients onto other professionals shows concern around ability to deal with this situation; that it was not their role to raise the issue, but they could respond. Several reported trying to create an environment that was conducive to such conversations, stressing the importance of making patients comfortable and then asking about broader topics, changes to home life, relationship, etc. As much as HCP27 did not identify herself as asking about sexual wellbeing, she is clearly providing opportunity for it to be raised.

I would always ask about life, I would always ask about relationships at home, I would always ask about work, I would always ask about social circumstances but – and I would always ask how your wife is coping or how your husband is coping and that side of things, but I wouldn't necessarily specifically ask about the sexual side of their relationship. I think if you do ask something that's very intimate and personal like that, I think it's important always to place it in context and not just ask it out of the blue. HCP27-Doctor

Difference in HCP perspective between acute care and care once patients left hospital seemed due to the acute versus holistic nature of their posts. The focus in acute settings was on patients' immediate survival needs, whereas later on it was possible to deal with a wider range. HCPs later on seemed less shocked with the idea that patients might want to discuss sexual wellbeing.

#### Patient level

At the level of the patient, a minority of HCPs thought that sexual wellbeing would not be relevant to patients at all. More common was the view that it was not a priority for patients or indeed the staff, particularly during the acute phase where getting back to basic health was the aspiration. Furthermore there was a feeling that patients would not be aware of sexual wellbeing as a potential problem whilst in hospital, as they would not be having or thinking about sex at that point.

HCP5: Maybe that could be later down the road. Maybe, rather than in the first six weeks talking about that.

HCP3: Depends what your priorities are [name of HCP5]? [laughs]

HCP4: Yeah, to eat, drink and look after yourself is kind of a higher priority generally.

HCP3, 4 &5-Therapists

Later on in care, there could still be more pressing matters, with emphasis on the ‘medical model’, including blood pressure and medications. Even in the context of sexual wellbeing, problems were perceived to be mechanical for example diagnose erectile dysfunction and prescribe medication to solve. Some HCPs acknowledged the emotional side, but then were less certain how to deal with it.

I mean it wouldn't be top of my list to be honest, you know. I sort of think making sure that they're on the right medication, that the blood pressure's well controlled, that they're medically stable would be my number one thing really. Then, you know, perhaps advise them about driving would – you need to work out just how they're coping at home and I guess that it would fall under the coping at home category really… If it was a man who was like struggling with like erectile dysfunction or something then I'd either consider suitability for Viagra or I'd refer onto an urologist and kind of let them deal with it really HCP28-Doctor

One participant voiced concern about raising the issue that it could indeed cause harm to the patient, for example mentioning sexual dysfunction might have a psychological effect and result in increasing the likelihood of developing dysfunction.

I have had a few people come back and say, you know ‘I've noticed this [erectile dysfunction]’. I've said ‘Well this could be your medication.’ But it's not the first thing I say when I start issuing this medication. Because I'm not sure whether it has a psychological component as to whether it will affect their performance if I tell them that ‘this could affect your performance’. HCP9-Doctor

#### HCP-Patient interface

At the HCP-patient interface across the pathway and within each professional group, there were individuals who reported concern that raising the issue of sexual wellbeing could harm the relationship. It was felt there was potential to embarrass or offend the patient or fear that the patient would think badly of the HCP for raising the issue.

*I: Would you ever raise it, do you think?*

HCP16: I don't think I would, only because it's quite a sensitive thing, because at the end of the day, once, when a patient comes in new, they're a stranger to you, aren't they, and it's quite a personal thing to bring up with somebody, and a lot of people get embarrassed. You have to be quite careful at how you approach certain things. So, I don't know, I don't feel equipped to be able to do that. HCP16-Nurse

Interestingly there was some debate about whether a long term relationship between HCP and patient would either support or inhibit such conversations and whether the relative anonymity of someone they know less well might provide a safer space. Furthermore there was discussion as to whether the role of the HCP, or other characteristics such as gender, influence the likelihood of asking: would a patient be more comfortable with a nurse (often female) whom they have more contact with or a doctor (either male or female) whom they may feel would have more expert knowledge.

For those participants who had experience of discussing sexual wellbeing with patients, there was awareness that patients may not want partners present for discussions. Those who had had such experiences dealt with them in different ways, either they accepted the request and talked to patients on their own, or in one instance as described by HCP12, she insisted on a joint discussion with both partners. It was felt inappropriate to raise discussion of sexual wellbeing if there were other family members present.

[The patient] actually asked me in a room away [from partner], so I had to bring them back in because it, it was obvious they both had to talk. But not being a sexual counsellor in any type of thing, I was ‘gaww’, I was thinking ‘Get me out of here.’ But professionally anyway it worked. HCP12-Support Coordinator

Raising the issue of sexual wellbeing could imply that patients had been having sex and wanted to have sex again in the future. One HCP, HCP30, reported experience of having such discussions being unwelcome as stroke could be viewed as an excuse by a partner to stop engaging in sexual intercourse.

I've seen it has been sort of its sort of men being told [by partners] they shouldn't have [sex] because they've had a stroke, and when that's been, you know, when I've said ‘no’, that's clearly that's been a - that's been the wrong thing to say. HCP30-Doctor

HCPs suggested characteristics of patients they felt it would be inappropriate to discuss with, primarily people who they thought would not be having sex: those who live alone or in a nursing home, were widowed, cognitively impaired or more broadly ‘older’. Despite their being acknowledgement amongst some interviewees that older people have sex, there were more extreme views presented such as HCP20.

Obviously, a lot of our elderly gentlemen and ladies don't do that [have sex] anymore, because their partners have either passed away or they don't think it's suitable … the older generation tend to stick to one partner. HCP20-Health Care Assistant

In addition there were a minority of HCPs who perceived sexual wellbeing to be not worth raising. This was either for young people because there was an assumption it ‘goes back to normal’. Or for women as there was the perspective that a stroke would be unlikely to affect their sexual wellbeing or indeed that they are less interested in sex in any case.

A man might want to know that he can still perform in that way. It doesn't matter so much for a woman because she doesn't have to…a woman, when I say it doesn't matter, she hasn't got to have an erection, has she? She hasn't got to actually…a woman can have sex without actually doing anything physical if you like. HCP26-Support Coordinator

### Resources available but underutilised

Many HCPs were unaware of the existence of the Stroke Association ‘Sex after stroke’ information leaflet, despite both hospitals having Stroke Association representatives (Support Coordinators) visiting patients weekly on the stroke wards.

*I: Have you ever seen any information about that about, or…?*

HCP18: No, I haven't. I know we've got the stroke booklet but I haven't had a look at it. I'm not sure really what it's got in it. From a doctor's stroke guidelines, it's more about dealing with acute situations. So, no, I've not seen any leaflets, nothing like that. HCP18-Doctor

However, even for those who did know of their existence use of such information was not routine. Support coordinators have a tool which outlines a range of potential discussion topics to raise with patients who have had a stroke and their carers, with sexual health as one of them. However this was seen as optional and reported frequency of discussion varied by support coordinator. Strategies were employed to avoid directly raising the issue: waiting for patients to raise it; highlighting there are a range of information sheets, giving patients responsibility for selecting that specific sheet and giving the sheet out in a bundle along with many other sheets. The first two strategies continued to put the impetus on the patient.

I remember giving someone a couple of fact sheets. And I said ‘Well actually, you know, this is the list that, that I've got’ I said ‘is there anything else that you actually want?’ And that was the one they chose. So it wouldn't have been in my thought process to say something about it straight away. So it was a way forward anyway… HCP11-Support Coordinator

Even the most frequent discussant of sexual wellbeing post-stroke, who gave leaflets out quite frequently, still used her discretion when deciding who to give them to. Her ability to routinely hand out ‘Sex after stroke’ leaflets seemed grounded in her view that it was an area of importance for people, believing that patients might want to discuss it even if they had not raised it, which was evidenced by her experience of having had ‘a number of people’ talk about it. She also reported having either neutral or positive responses to distributing the leaflet, so had not been put off distribution. She was confident about the boundaries within which she could discuss it, and reported having referred patients to their GP. She reported that patients were comfortable with her, citing that they knew she was there to support them, and a certain trust had been built as she was seeing them across their patient pathway.

I'm going through my papers and say “oh, perhaps you could, might like to lose a little bit of weight so I'm putting this healthy eating one in” and then I'll say “and there's a ‘Sex After Stroke’ and I'm putting that in” and they just usually say thank you. Then sometimes they might say “Oh, we never had any sex before the stroke [Laughter] so why should we have it after” and they make a joke of it. HCP26-Support Coordinator

## Discussion

### Main findings

Most HCPs interviewed in this study perceived significant barriers to initiating discussion about sexual wellbeing leaving the onus on patients to raise it. Issues concerning role and perceived competence were driven by minimal training regarding sexual issues leading to a lack of responsibility for it on the part of HCPs in general and little consideration of it within the stroke care pathway in particular. Only stroke support coordinators reported having it specified in their remit.

Many HCPs considered it inappropriate to raise sexual wellbeing as a discussion topic and that doing so could harm the clinician/patient relationship. HCPs were unaware of relevant patient information sources or underutilised them. Barriers to information provision included concerns regarding how to sensitively communicate sexual topics, for example concerns over raising the issue in front of a patient's partner. An additional factor was HCPs' understanding of who the appropriate recipients of such information would be, for example their relevance to people who are ‘older’. The minority of HCPs who reported confidently having such discussions suggest that normalisation of the consideration of sexual wellbeing could be possible.

### Strengths and Limitations of the study

The key strengths of this study were the range of HCPs included and that sexual wellbeing was one component of a broader interview on experience of the stroke patient pathway. Therefore, participants who might not have volunteered for an interview specifically on sexual wellbeing were able to talk about it in context. The ability to interview HCPs involved at different stages along the patient pathway ensured we could examine what patients might experience throughout their journey after stroke. RM conducted all HCP interviews; she is a non-clinician and informed participants of this during the interviews. This non-clinical background may have influenced how or what participants reported, and enabled the interviewer to probe participants as an outsider within a “safe” context. The majority of participants were interviewed on their own, but a few chose to be interviewed alongside close colleagues. Discussion of sensitive topics in front of a colleague has the potential to inhibit discussion, due to fear of repercussions or gossip[46] but this did not appear to be the case: length and depth of discussion were similar to individual interviews and in some cases the participants' interaction stimulated further discussion.

A limitations of this study is the small proportion of male HCPs interviewed, although this is representative of the NHS workforce[47]. In addition, interviews were conducted in HCPs' places of work, with limited time available and in a few instances were ended prematurely due to HCPs being called away. The experiences recounted may have been influenced by the fact that all participants were from one area of the country and we cannot comment on how similar or widely these views are shared among the wider HCP community.

### Comparison with the literature

The current study presents novel data in stroke but similar barriers have been previously reported in primary care[23], [24], a mental health ward[48], coronary heart disease treatment[23] and cancer treatment facilities[19]–[21]. Lack of time, of privacy in ward setting, and of emphasis in guidelines have all been described. As with stroke, cancer studies have suggested that sexual wellbeing is not the main priority of the patients during treatment[19], [20]. This often emerges as a patient need at some point during the recovery period but generally remains unaddressed because patients lack knowledge of how to access care and receive not encouragement to do so from HCPs [49]. Many patients are simply embarrassed about raising the issue of sexual wellbeing, lack confidence or perceive help-seeking as too risky [50].

Widespread reporting of stereotyping around patients' age, gender and ethnic background suggests an endemic problem within health services in general [19]–[21]. Parallels can be drawn with other areas of patient care including faecal and urinary incontinence, where stigma appears to preclude access to effective treatment[51]. Up to 75% of patients with faecal incontinence do not seek medical help[52] and finding appropriate information on these conditions can be difficult for patients[53] highlighting the important role of HCPs. Yet, a Canadian study found that only 35% of family physicians were comfortable dealing with urinary incontinence[54]. Unvoiced patient agendas often lead to misunderstandings, dissatisfaction and poor outcomes[55]. A European comparative study found that urinary incontinence care in the UK was considerably worse than elsewhere[56].

### Clinical implications

This study suggests that whilst structural changes may be needed – in terms of inclusion of sexual wellbeing in care pathways and improving information provision, relatively small changes might allow individual HCPs to change practice by legitimising the topic, and making its consideration routine practice[57]. This is important as having a stroke can impact on patients' and their partners' sexual wellbeing, be it sexual dysfunction or concerns over safety of having sex.

Increased awareness amongst HCPs that sexual wellbeing can be an area of importance for many people, regardless of gender or age might be achieved through simple training. Building communication skills around raising sensitive topics may also be of value to discussing other taboo areas including incontinence and could be tackled both pre- and post-qualification. Routine provision of information such as that provided by the Stroke Association could help HCPs know what resources and services are available. Further training on sexual health management has been called for previously[19], [21], [22], [24], [58]. This study suggests that recognition of the legitimacy of patients having sex lives, and the appropriateness of such discussions needs a proactive approach including explicit “permission” to raise sensitive topics along with time and a comfortable environment to have these discussions. Interventions that would facilitate this are not expensive and should be implementable in a wide range of settings.

Integrated care pathways are becoming more common within the NHS as a mechanism for managing clinical processes and improving patient outcomes [59]. Coordinating Primary and Secondary Care provision is increasingly necessary for the management of chronic conditions. Also, recognition of the importance of self-management support is growing [60]. These twin approaches based on coordinated care [61] and empowering patients are not unknown in the management of stroke [62]. Through a recent qualitative synthesis, *regaining or developing a new self and roles* has been identified as a major issue following stroke[63]. The need for focussed self-management programmes to address this broad issue of recovery and adjustment has been strongly suggested but the lack of specific attention to sexual wellbeing in this literature remains a matter of concern.

## Conclusion

Some HCPs lack motivation, ownership and the confidence and skills to raise sexual wellbeing routinely after stroke, potentially resulting in a sub-optimal experience for their patients. Normalisation of the inclusion of sensitive topics in discussions post-stroke does not seem to need large structural transformation; rather, simple changes such as information provision and acknowledgement of the issue in standard care policies. The experiences recounted by professionals in this study suggest that such policies require attention now.
